# The Law Relating to Infectious Diseases.—III

**Published:** 1902-08-16

**Authors:** J. E. R. Stephens

**Affiliations:** Barrister-at-law, Author of "Digest of Public Health Cases," etc. etc.


### THE INSTITUTIONAL WORKSHOP.

### THE LAW RELATING TO INFECTIOUS DISEASES.—III.

By J. E. R. Stephens, Barrister-at-law, Author of "Digest of Public Health Cases," etc. etc.

### Canal Boats.

By the Canal Boats Act, 1877, sect. 2, the Local Government Board may make regulations *inter alia*, for promoting cleanliness in and providing for the habitable condition of canal boats; and for preventing the spread of infectious disease by canal boats. The Local Government Board have made regulations under this section, dated March 20th, 1878. They provide *inter alia* as follows:— "(12) In every case where a person on a canal boat is seriously ill or is evidently suffering from an infectious disease, the master of the boat, if at the time the boat is proceeding on a journey shall, as soon as may be practicable, give information thereof to the sanitary authority within whose district is situated the canal or the part of the canal along which the boat may at the time be passing, and on the arrival of the boat at its port or place of destination, to the sanitary authority within whose district such port or place is situate, and also to the owner of the boat. If at the time the boat be at its port or place of destination, the master shall forthwith inform the sanitary authority within whose district such port or place is situate, and also the owner of the boat, that a person on board the boat is seriously ill or is evidently suffering from an infectious disease. The owner of the boat shall forthwith upon the receipt from the master of information to the effect that a person on board the boat is or has been suffering from an infectious disease, give notice to that effect to the sanitary authority having jurisdiction in the place to which the boat may have been registered as belonging. (13) In every case where, in the exercise of the power conferred by sect. 4 of the Canal Boats Act, 1877, a sanitary authority may have detained a canal boat for the cleansing and disinfection thereof, the authority, before allowing the boat to proceed on its journey, shall obtain from their medical officer of health, or from some other legally qualified practitioner, a certificate to the effect that the boat has been duly cleansed and disinfected, and shall cause such certificate to be delivered to the master of the boat. The sanitary authority may pay a reasonable remuneration for such certificate."

Sect. 4 of the Canal Boats Act, 1877, enacts that where any sanitary authority within whose district a canal or any part of a canal is situate is informed by the master of a canal boat or otherwise that a person on a canal boat is suffering from an infectious disorder, the authority shall cause such steps to be taken as may, by the certificate of their medical officer of health, or of any other legally qualified practitioner, appear requisite for preventing the said disorder from spreading, and for that purpose may exercise the power of removing a person suffering as aforesaid, and all other powers in relation to provisions against infection conferred by the Public Health Act, 1875, and may also, if need be, detain the boat; but such boat shall not be detained a longer time than is necessary for cleansing and disinfecting the same.

Sect. 5 enacts that where any person duly authorised by a registration or sanitary authority, or by a justice of the peace, has reasonable cause to suppose, either that there is any contravention of this Act on board a canal boat, or that there is on board a canal boat any person suffering from an infectious disorder, he may, on producing (if demanded) either a copy of his authorisation, purporting to be certified by the clerk or a member of the sanitary anthority, or some other sufficient evidence of his being authorised as aforesaid, enter by day such canal boat and examine the same and every part thereof, in order to ascertain whether on board such boat there is any contravention of this Act, or a person suffering from an infectious disorder, and may, if need be, detain the boat for the purpose, but for no longer time than is necessary. The master of the boat shall, if required by such person, produce to him the certificate of registry (if any) of the boat, and permit him to examine and copy the same, and shall furnish him with such assistance and means as such person may require for the purpose of his entry and examination of and departure from the boat in pursuance of this section. A refusal to comply with the requisition of such person under this section shall be deemed to be an obstruction of such person. If such person is obstructed in the performance of his duty under this Act in the case of any boat, the person so obstructing shall be liable to a fine not exceeding forty shillings. The expression "by day" means between the hours of 6 A.M. and 9 P.M.

### Common Lodging-Houses.

By sect. 80 of the Public Health Act, 1875, every local authority shall from time to time make by-laws: (1) For fixing and from time to time varying the number of lodgers who may be received into a common lodging-house, and for the separation of the sexes therein; and (2) For promoting cleanliness and ventilation in such houses; and (3) For the giving of notices and the taking precautions in the case of any infectious disease; and (4) Generally for the well-ordering of such houses.

By sect. 82 the keeper of a common lodging-house shall, to the satisfaction of the local authority, limewash the walls and ceilings thereof in the first week of each of the months of April and October in every year, and shall, if he fails to do so, be liable to a penalty not exceeding 40s.

Sect. 84 enacts that the keeper of a common lodging-house shall, when a person in such house is ill of fever or any infectious disease, give immediate notice thereof to the medical officer of health of the local authority, and also to the Poor Law relieving officer of the union or parish in which the common lodging-house is situated. See also the Infectious Diseases (Notification) Act, 1889.

Sect. 85 enacts that the keeper of a common lodging-house and every other person having or acting in the care or management thereof, shall, at all times when required by any officer of the local authority, give him free access to such house or any part thereof, and any such keeper or person who refuses such access shall be liable to a penalty not exceeding £5.

Any keeper of a common lodging-house who, *inter alia*, fails to give the notices required by this Act, where any person has been confined to his bed in such house by fever or other infectious disease, shall be liable to a penalty not exceeding £5, and in the case of a continuing offence, to a further penalty not exceeding 40s. for every day during which the offence continues. (Sect. 86, sub-sect. 3.)

In any urban or rural sanitary district in which Part III. of the Public Health Acts Amendment Act, 1890, has been adopted, any keeper of a common lodging-house who fails to give the notice required by sect. 84 of the Act of 1875, is liable to a penalty not exceeding 40s., and to a daily penalty not exceeding 5s.

The Local Government Board have issued a series of model by-laws for the purposes of sect. 80. These by-laws prohibit the reception in any common lodging-house or room therein of a greater number of lodgers than the maximum from time to time fixed by the sanitary authority by a notice served on the keeper of the lodging-house, and provide for the separation of the sexes in rooms used as sleeping apartments. They require every yard or other open space within the curtilage of the premises to be maintained in good order and thoroughly cleansed. They also provide for the cleansing of the floors, windows, fixtures, painted surfaces, bedding, and bed-clothes of the lodging-house, the proper supply of basins and towels for the lodgers, the removal of all solid or liquid filth and refuse, and the cleansing of vessels and utensils before ten o'clock every morning; the keeping of the water-closets, drains, earth closets, privies and ashpits in good order and efficient action and in a wholesome condition; the maintenance of all means of ventilation in proper order; the keeping open for an hour every morning and afternoon of the windows of every room used as a sleeping apartment except in cases where the state of the weather renders it necessary for the windows to be closed or the room is occupied by a sick person; and the exposure to the air for an hour in the morning or afternoon of the bed-clothes after they have been used. They require every keeper of a common lodging-house immediately after he has been informed or has ascertained that any lodger is ill of any infectious disease to adopt all such precautions as may be necessary to prevent the spread of the disease, and prohibit him from at any time, while such lodger is suffering from such disease, allowing any other person, except the wife or other relatives of the lodger, or a person voluntarily in attendance on him, to occupy the same room. Where in pursuance of the statutory provision on this behalf (*i.e.* sect. 124 of the Public Health Act 1875) the sanitary authority order the removal of such lodger to a hospital or other place for the reception of the sick, they require the keeper on being informed of the order to forthwith take all such steps as may be requisite on his part to secure the safe and prompt removal of the lodger in compliance with the order, and in and about such removal to adopt all such precautions as, in accordance with any instructions which he may receive from his medical officer of health, may be most suitable for the circumstances of the case. Where, in consequence of the illness of the lodger, there may be reasonable grounds for apprehending the spread of infection through the admission of lodgers to any room or rooms in such house, or through the admission to such room or rooms of the maximum number of lodgers authorised to be received therein, the by-laws require the keeper, after being furnished with the necessary directions from the medical officer of health, and until the grounds for apprehending the spread of infection have been removed, to cease to receive any lodger in such room or rooms, or to receive therein such number of lodgers, being less than the maximum number, as the exigencies of the case require. Immediately after the death, removal, or recovery of any lodger who has been ill of infectious disease, they require the keeper to give notice thereof to the medical officer of health, and as soon as conveniently may be to cause every part of the room which may have been occupied by the lodger to be thoroughly cleansed and disinfected, and to cause every article therein which may be liable to retain infection to be in like manner cleansed and disinfected, unless the sanitary authority have ordered it to be destroyed. They further require the keeper to comply with all instructions of the medical officer of health as to the proper cleansing and disinfection of the room and articles, and to give written notice to the medical officer of health when the same have been thoroughly cleansed and disinfected in accordance with his instructions, and until two days have elapsed from the giving of this notice, and until the necessary precautions for preventing the spread of disease have been duly taken, they prohibit the keeper from allowing any other lodger to be received into the room which has been thus exposed to infection.

